# Technology to Improve Autonomy and Inform Housing Decisions for Older Adults With Memory Problems Who Live at Home in Canada, Sweden, and the Netherlands: Protocol for a Multipronged Mixed Methods Study

**DOI:** 10.2196/19244

**Published:** 2021-01-21

**Authors:** Jodi Sturge, Louise Meijering, C Allyson Jones, Mirjam Garvelink, Danielle Caron, Susanna Nordin, Marie Elf, France Légaré

**Affiliations:** 1 Population Research Centre Faculty of Spatial Sciences University of Groningen Groningen Netherlands; 2 Faculty of Rehabilitation Medicine University of Alberta Edmonton, AB Canada; 3 VITAM - Centre de recherche en santé durable Quebec, QC Canada; 4 School of Education, Health and Social Studies Dalarna University Falun Sweden; 5 VITAM - Centre de recherche en santé durable, CIUSSS de la Capitale-Nationale Department of Family Medicine and Emergency Medicine Université Laval Quebec City, QC Canada

**Keywords:** aging in place, co-design, cross-country comparison, electronic decision support intervention, housing decisions, memory problems, mixed methods, mobility patterns, shared decision making, technology

## Abstract

**Background:**

Understanding the mobility patterns and experiences of older adults with memory problems living at home has the potential to improve autonomy and inform shared decision making (SDM) about their housing options.

**Objective:**

We aim to (1) assess the mobility patterns and experiences of older adults with memory problems, (2) co-design an electronic decision support intervention (e-DSI) that integrates users’ mobility patterns and experiences, (3) explore their intention to use an e-DSI to support autonomy at home, and (4) inform future SDM processes about housing options.

**Methods:**

Informed by the Good Reporting of A Mixed Methods Study (GRAMMS) reporting guidelines, we will conduct a 3-year, multipronged mixed methods study in Canada, Sweden, and the Netherlands. For Phase 1, we will recruit a convenience sample of 20 older adults living at home with memory problems from clinical and community settings in each country, for a total of 60 participants. We will ask participants to record their mobility patterns outside their home for 14 days using a GPS tracker and a travel diary; in addition, we will conduct a walking interview and a final debrief interview after 14 days. For Phase 2, referring to results from the first phase, we will conduct one user-centered co-design process per country with older adults with memory issues, caregivers, health care professionals, and information technology representatives informed by the Double Diamond method. We will ask participants how personalized information about mobility patterns and experiences could be added to an existing e-DSI and how this information could inform SDM about housing options. For Phase 3, using online web-based surveys, we will invite 210 older adults with memory problems and/or their caregivers, split equally across the three countries, to use the e-DSI and provide feedback on its strengths and limitations. Finally, in Phase 4, we will triangulate and compare data from all phases and countries to inform a stakeholder meeting where an action plan will be developed.

**Results:**

The study opened for recruitment in the Netherlands in November 2018 and in Canada and Sweden in December 2019. Data collection will be completed by April 2021.

**Conclusions:**

This project will explore how e-DSIs can integrate the mobility patterns and mobility experiences of older adults with memory problems in three countries, improve older adults’ autonomy, and, ultimately, inform SDM about housing options.

**Trial Registration:**

ClinicalTrials.gov NCT04267484; https://clinicaltrials.gov/ct2/show/NCT04267484

**International Registered Report Identifier (IRRID):**

DERR1-10.2196/19244

## Introduction

### Background

Estimates of life expectancy are increasing, yet age-related disability is still not decreasing [1,2]. Cognitive impairment, including memory problems, are increasingly considered a major public health, economic, social, and political challenge. Projected estimates are that over 75 million older adults worldwide will experience some form of dementia by 2030 [3]. Most older adults with dementia want to remain at home, in a familiar environment, as long as possible [4,5]. For those people with dementia who live at home, engaging in activities outside the home can predict vulnerability. For instance, one study found that 43% of people with dementia wander at some point [6]. Not all people with dementia, especially at the early stage of the disease, have a formal diagnosis or may feel stigmatized by the word [7-9]. Therefore, for the purpose of this research, the term “memory problems” will be used to identify participants rather than dementia. Further, in this protocol we refer to “housing decisions” as decisions about how to remain safely at home as well as decisions about whether to move elsewhere or not. Memory problems and other dementia-related behaviors, along with a need for more skilled health care services, can be a strong incentive to relocate people with dementia to residential care facilities, even against their will [10,11]. Whether to stay at home (ie, age in place) or move to a residential care facility is a frequent, and difficult, decision for older adults with health and memory problems and their informal and formal caregivers [12]. Moreover, the decision about whether it is time to relocate may have to be faced repeatedly as autonomy diminishes and the current housing situation may not provide enough support. Older adults with memory problems may express the desire to stay at home [5] but they and their caregivers are often unaware of options available to them that could support them to remain safely at home. Furthermore, older adults with memory problems are often unaware of their right to be engaged in the decision-making process, which undermines the quality of housing decisions for them and their caregivers [13-15].

New technologies could extend the period of time during which older adults with memory problems can remain mobile and autonomous [16]. Alarm bracelets with communication systems, assistive walking devices, support bars, and adaptive kitchen equipment are commonly used to support independence in indoor environments [17,18]. There is enormous potential for other technological interventions that are used extensively in other sectors, such as GPS tracking, indoor beacon technology, bed sensors, and personal activity monitors to measure movement (ie, acceleration, cadence, and steps) [19,20], detect falls [21], and measure activity inside and outside the home or body metrics (eg, blood pressure and heart rhythm) [22]. These technologies could be enlisted to support mobility and autonomy in the home environment and hold great potential for older adults whose informed value-congruent decision is to stay home [23-26]. However, few technologies have been applied or tested with older adults in real-life settings outside of residential care facilities. Further, there have been limited attempts to use technologies to assist older adults with memory problems to stay at home longer, and data from such devices have not been used to inform housing decisions. This study builds on our current work [27,28].

To improve the quality of housing decisions among older adults with memory problems, team members of this study have performed systematic reviews [14,29] and created and evaluated electronic decision support interventions (e-DSIs) to inform and foster shared decision making (SDM) [14,30]. However, few studies have examined the relationship between the dynamics of mobility patterns and mobility experiences outside the home and how an understanding of mobility can inform SDM about housing options. Person-centered care promotes patient autonomy, empowerment, and value-congruent choice [31,32] and is key to the next generation of health reforms [33]. SDM is the cornerstone of person-centered care and a process whereby the people involved in decision making identify the decision to be made and discuss risks and benefits of the options, as well as the preferences of all involved [15,34-37]. In the case of older adults with memory problems and their caregivers, SDM requires conveying information in a way that will engage them in the decision-making process by helping to clarify what matters most to them. Preferred options need to be assessed and, if possible, tailored to meet the person’s understanding and needs. Decisions made in this way increase satisfaction, increase adherence to decisions made, and decrease decisional regret [38]. SDM is especially important in preference-sensitive decisions or in circumstances where decision making is plagued with uncertainty, such as housing decisions [29]. Housing decisions for older adults facing loss of autonomy are distinct in that their level of autonomy related to memory problems may be changing and their decisions can quickly be out of date. Thus, the decision-making process may need to be repeated and requires updated information on a regular basis. We hypothesize that an adapted e-DSI and the devices connected to it could play an important role in informing and updating this decision.

### Objectives

The overall aim of this research project is to understand the mobility patterns and experiences of older adults with memory problems living at home and how this data can improve autonomy and inform SDM about housing options. Our multidisciplinary and international team aims to address the following research questions: (1) What are the mobility patterns and mobility experiences of older adults with memory problems? (2) How can an e-DSI be co-designed and adapted to integrate mobility patterns to improve autonomy? (3) What is the intention among older adults to use the e-DSI for future decision making about housing? and (4) How can this research inform SDM processes about housing decisions?

## Methods

### Overview of Study Design

The COORDINATEs (teChnology tO suppORt DecIsioN making about Aging aT homE) project is a multipronged study with four phases, each one under the leadership of team members in a different country. Our research questions will be addressed in three countries, with a diversified sample, using a mixed methods approach to provide rich insight into mobility patterns and decision making. Using an integrated knowledge translation approach, we will use iterative end-user consultation and feedback to tailor e-DSI technology to end users in Canada, Sweden, and the Netherlands.

Canada, Sweden, and the Netherlands all are advanced economies with welfare states with aging populations. However, each country has a different health and welfare system that influences policies and services dedicated to aging at home versus in institutions [39,40]. Canada’s regime is liberal with a national health insurance system, while Sweden has a social-democratic welfare regime. The Netherlands has characteristics of both conservative and social-democratic regimes and an etatist social health insurance system. We will take into account the intra- and intercountry differences between older adults and between urban and rural environments. Data collection for each phase will be carried out simultaneously and in collaboration with all country teams. The target population and recruitment strategy will vary for the three data collection phases (see Table 1). Phase 1 will be under the leadership of Dutch team members and will assess mobility patterns and experiences of older adults with memory problems in all three countries. Phase 2 will be under the leadership of a Swedish team and will support the co-design of an e-DSI that integrates mobility patterns and experiences generated from Phase 1 and end users’ views and preferences for improving the usability and adaptability of technology [41]. Phase 3 will be under the leadership of Canadian team members who will oversee web surveys—one in each country—on the end users’ intention to use personalized e-DSIs to improve autonomy and inform housing decisions. Lastly, for Phase 4, the leadership in all three countries will triangulate and compare results to develop an action plan for scaling up e-DSI development to improve autonomy among older adults with memory problems who live at home and inform their SDM processes about housing options. Informed by the Good Reporting of A Mixed Methods Study (GRAMMS) reporting guidelines [42], and applying an integrated knowledge translation approach [43], we will explore synergies between the data, inform the design of the sequential phases, and link the findings.

**Table 1:** Target population and recruitment strategy: Phases 1 to 3.

Project phase	Target population	Recruitment strategy
Phase 1	Older adults with self-reported memory problems who live at home	Referrals from nurses and physicians Referrals from home health care teams Self-referrals from flyers in public spaces Self-referrals from media
Phase 2	Older adults with self-reported memory problems Caregivers Individuals with experience providing care to a person with memory issues Health care professionals Policy makers Information technology representatives	Network of the research team Reference group representing older people
Phase 3	Older adults with self-reported memory problems Caregivers Health care professionals	A survey firm in each country will recruit participants through web panels

### Coordination and Management

An executive committee will oversee the operationalization of the project. The committee consists of the nominated principal investigator (FL), all coprincipal investigators in each country (AJ, LM, and ME), two representatives of older adults and caregivers (to be determined), one trainee (JS), and one research coordinator (DC). A steering committee consisting of team members and partners will oversee the development and implementation of all phases of the study and will meet annually in person or virtually. In accordance with the funding agencies, our research team will attend the annual meetings of the project’s funding agency, the More Years, Better Lives consortium [44].

### Phase 1: Mobility Patterns and Experiences

#### Participants

In each country, convenience samples of 20 older adults living at home with memory problems will be recruited using several methods, including distribution of flyers in the community and at health service sites, referrals from community physicians, home health care teams, and media. The research teams will also provide a project overview presentation to local dementia case management teams as a way to elicit suitable referrals. Inclusion criteria for this phase of the study are (1) being over the age of 65 years; (2) living at home independently with a partner, family member, or alone; (3) and experiencing memory problems. Participants will be asked to self-identify as experiencing memory problems, and the severity of the problems will not be assessed by the research team. Our population target is older adults who live at home independently with a partner, family member, or alone. Thus, it is not necessary to have a caregiver to participate in this study. Although caregivers are not the target population for this phase of the research, if the research participant requests it, the caregiver will be asked to share their own personal experiences and to liaise between researchers and older adults if necessary. In situations where caregivers participate in the research, they will be asked to sign a consent form. We aim for a diverse group of participants (eg, sex, health status, and geographic region). Our mixed methods approach is well-suited to gain in-depth insight into the mobility patterns and experiences of older adults experiencing memory problems.

#### Data Collection

Data will be collected by using (1) a sociodemographic survey, (2) a walking interview, (3) GPS tracking, (4) travel diary entries, and (5) an in-depth interview (see Table 2). Each method provides unique data and, in combination, the data provide a comprehensive overview of mobility. For instance, the quantitative data collected through the survey will be analyzed to describe the sample. The qualitative data from the walking and debrief interviews will provide context for the mobility experiences, and the GPS data will provide insight into the spatial mobility patterns. While there may be gaps in the GPS data related to poor connectivity or if the participant forgets to take the GPS tracker with them to an activity, as seen in other studies with older adults and GPS data [28], the travel diary will be used to provide insight into the missing data.

**Table 2:** Data collection methods to assess mobility patterns and experiences.

Method	Description
Sociodemographic survey	A 21-item Likert scale-based questionnaire that includes questions on sociodemographics, living environment, social activities, and standardized self-rated health questions will be administered.
Walking interview	Participants will be asked to take the researcher on a walk of a typical route they take near their home. Throughout this participant-led interview, the researcher will ask questions about typical experiences the participants have when taking the route.
GPS tracking	We will provide participants with a GPS tracking device (eg, Qstarz BT-1000X) to track the routes and location of activities for a period of 14 days. GPS trackers collect spatial data, consisting of latitude, longitude, date, and time, in 5-second epochs.
Travel diary	To complement the GPS data, the participants will be asked to record daily activity information in a formatted travel diary. Activity information includes date, day of the week, time of departure, arrival time, location, purpose of the activity, mode of transportation, person with whom they traveled and did the activity, the use of a mobility aid, and whether the activity was planned.
Debrief interview	After 2 weeks of data collection, an audio-recorded debrief interview will be scheduled to review the participant’s activities and mobility experiences.

The research team will carefully explain the data collection and storage process through oral and written information. The participants will then be asked to sign a consent form before the data collection begins. A research assistant will conduct the walking interviews with the participants to generate insights into the participants’ movements in their neighborhoods [45]. The research assistant will walk along with each older adult and ask questions along the way, thereby capturing rich data on attitudes and feelings about their environment, special practices, social architecture, and biographies [46,47]. After 2 weeks of data collection, we will conduct debrief interviews to discuss participants’ mobility patterns and experiences in relation to their self-reported well-being [16,28]. This last interview will more generally explore the participants’ mobility experiences and motives for staying at home in their neighborhood.

#### Data Analysis

In line with other studies using similar methods, the sample size of 20 participating older adults per country is based on the expectation that it should reach data saturation (eg, [28,48,49]). A database that includes quantitative data from travel diaries will be created in Microsoft Access 2016, and the data will be cleaned, analyzed descriptively, and linked to the GPS data based on unique identifiers. All data, aside from the GPS data, will be anonymized and pseudonymized. The GPS data are extremely privacy-sensitive as they provide location information, so they will be securely stored in a virtual research workspace (VRW) created by the Center for Information Technology at the University of Groningen. Only project staff can access this environment via two-factor authentication (ie, password and text message). When analyzing the GPS data, we will work with the real location data within the VRW. The real locations are needed as the location and movement data need to be connected to other layers of information, such as roads, shops, and health care services. GPS data will be analyzed using the geographic information system programs V-Analytics and ArcMap 10.5.1 (Esri) to create maps with visited places and trajectories, including speed, duration, and length of time on an activity. The qualitative data generated through the walking interviews and debrief interviews will be transcribed verbatim, open contentcoded using a grounded theory method [50], and analyzed thematically using the software package ATLAS.ti 8.4 (Scientific Software Development GmbH). All data sources will be compared and combined to check for incongruences between the data sources to obtain a comprehensive overview of the participants’ mobility in association with their respective environments. Furthermore, we will summarize the themes from the in-depth interviews, translate them into English, triangulate data from the three countries, and further compare differences between health care systems, geography, and regulations.

### Phase 2: Co-Design of an Electronic Decision Support Intervention

#### Participants in the Co-Design Process

Using the research team members’ extensive networks, we will recruit a convenience sample of 5 to 8 end users per country that will include (1) older adults, (2) caregivers, (3) professionals with experience caring for people with memory issues, and (4) technology developers. The group size and configuration is based on other co-design studies [51]. We will strive to have an equal proportion of each type of end user with a group size of 8 persons. The inclusion criteria for the older adults will be individuals over the age of 65 years with memory problems, who receive support at home from health services, and who have access to a computer and the internet at home.

#### Data Collection

Based on data from Phase 1, we will conduct user-centered co-design workshops with older adults with memory problems and stakeholders. The aim is to co-design an adapted version of an existing e-DSI that could be used to improve autonomy and inform shared decision making for older people in frail health living at home, and to evaluate the co-design process. We will use the Double Diamond method [52] as a guide for the workshop facilitation. The Double Diamond method includes a three-step process: (1) idea generation, (2) modeling a prototype, and (3) testing and consensus discussions. Each country will facilitate their own workshops in keeping with the co-design approach.

#### Data Analysis

The size of the groups is adequate for a user-centered process [53], ensuring optimal participation of the end users in the co-design group. The qualitative data generated through the group discussion will be transcribed verbatim, open content-coded, and analyzed thematically using the software package ATLAS.ti 8.4. We will compare data from the three working groups and try to find common ground with regard to the solutions mentioned. Based on the findings, we will develop recommendations as to how to integrate mobility patterns into an e-DSI and we will upload interactive video-based material developed in a previous study [54] with information about further options for staying independent at home.

### Phase 3: Exploring the Intention to Use an Electronic Decision Support Intervention

#### Participants

We will hire a survey firm in each country that will recruit 70 older adults and/or caregivers. A total of 210 participants will be recruited from the survey firm’s web panels and via end-user organizations, such as senior and caregiver associations. Inclusion criteria include the following: (1) older adult who is 65 years old or older or a caregiver of an eligible older adult, (2) older adult who self-identifies as having memory problems, and (3) older adult who has access to a computer with internet access. Facing a housing decision will not be an inclusion criterion, but participants will be asked if this is a current decision-making process they face. We have determined our sample size based on the mean behavioral intention taken from Delanoë et al [55]. With a power of 80% and an α level set at .05, our survey will be able to detect a mean score of 5.6 (SD 0.14), 19 times out of 20 (margin of error equal to 0.14), within each country.

#### Data Collection

For each country, based on sociocognitive behavioral change theories such as the theory of planned behavior [56], we will assess participants’ opinions regarding factors [57] that could influence their adoption and intention to use an e-DSI to improve autonomy and make decisions about housing [13]. Participants will be invited to complete a self-administered web-based survey with closed-ended questions. The survey questions will be based on the integrative model of behavior [58] to assess participants’ intention to use the e-DSI for future if they faced a housing decision [13]. Additionally, we will measure the psychosocial determinants of this behavioral intention [14] and explore their preferred role in decision making [59], their experience using technology [13], and sociodemographic variables (eg, age, sex, social economic status, and relationship between the older adult and the caregiver) [57,60].

#### Data Analysis

Our primary outcome variable is the behavioral intention of a participant to use the e-DSI; this will be measured on a 7-point Likert scale, ranging from 1 to 7. We will assess the psychosocial determinants of this behavioral intention and participants’ opinions regarding factors that could influence their use of an e-DSI by referring to questions in the domains of the NASSS (nonadoption, abandonment, scale-up, spread, and sustainability) framework [61]. We will compare data from people with and without experience using an e-DSI to explore how experience influences willingness to use it. Secondly, we will compare the data obtained in the three countries to identify any differences between health care systems, geography, and regulations.

### Phase 4: Inform Future SDM Processes

For the final phase of the project, we will synthesize, triangulate, and compare data from all project phases and collaboratively explore privacy issues. Validation and interpretation of data will be accomplished with consensus meetings between researchers and end users [62]. Data from the three countries—Canada, Sweden, and the Netherlands—will be triangulated and compared to assess differences in contextual factors (ie, health care system, policy, geography, and culture), and we will make recommendations for technology implementation to improve autonomy and inform SDM about housing in the three countries. At the end of Phase 4, end users in each country will participate in a workshop where project results will be presented and discussed. As an output, we will acquire knowledge about differences and similarities in mobility patterns and experiences with using GPS and an e-DSI among older adults with memory problems and their caregivers, their respective assessments of its contribution to improving autonomy and emerging housing decisions, their willingness to continue using it, and factors that influence usage. After evaluating the impact of an e-DSI for continuing to age at home and for ongoing housing decisions among older adults and their caregivers, we will propose country-specific action plans to scale up e-DSIs and evaluate their implementation by home care services. We will explore opportunities to continue the consortium and form an infrastructure for continuous collaboration between the three countries.

### Ethical Considerations and Trial Registration

A review for issues regarding human subjects has been obtained from four research ethics committees. Ethics approval has been obtained by the Research Ethics Committee, Faculty of Spatial Sciences, University of Groningen; Centre intégré universitaire de santé et de services sociaux de la Capitale-Nationale, L'Université Laval; the Swedish Ethical Review Authority; and the Health Research Ethics Board, University of Alberta. For all phases of data collection, participants will be asked to sign an informed consent form. Where appropriate, we will ask informal caregivers to sign a consent form. This trial was registered at ClinicalTrials.gov (NCT04267484).

## Results

The study opened for recruitment in the Netherlands in November 2018 and in Canada and Sweden in December 2019. Data collection will be completed by April 2021. Given the COVID-19 pandemic and the different lockdown approaches taken by each country, data collection may be adapted using virtual methods.

## Discussion

We have described the methods for exploring how adding data about personal mobility patterns of older adults to an e-DSI has the potential to improve their autonomy and inform future SDM processes about housing options. Participants included older adults living at home with memory problems and their caregivers as well as a diverse group of stakeholders. This study will contribute to the mobility literature on older adults with memory problems and inform the development of technology that augments self-management among older adults affected by memory loss, their caregivers, health care professionals, and policy makers [13,14,16,30,60].

The proposed study is a highly original approach to the potential of technology use with older people with memory problems. First, this is an interdisciplinary, interprofessional, intersectorial, and international study. This question is often addressed in isolated contexts without the mutually enriching possibilities of working together with other disciplines, professions, technological traditions, and cultures. Second, while personalized medical care such as gene testing or drug treatment selection is becoming the norm in specific contexts, personalized data have rarely been used to empower older people with memory loss. The results not only have the potential to keep older people safely at home for longer but will provide deep insights into their physical and emotional relationship with their surroundings and the consequences of displacing them into a new environment. Third, one of the persistent problems we have seen with decision making about housing among older adults is that their autonomy or mobility needs change from day to day. Thus, the information needed for decision making also changes from day to day. This technology could relieve the deep distress experienced by older people and their families facing this decision by providing reliable and relevant ongoing data that indicate the individual’s changing needs for on-the-spot decision making.

Finally, new technologies such as GPS tracking can infringe on people’s privacy, an issue that is highly relevant today. For example, more people are using apps that provide contact tracing for people infected with COVID-19. In our cross-country comparisons, we will investigate the ethical issues involved in working with tracking technologies with older adults in the three countries. There is little research on the ethical implications of these technologies with older adults and their caregivers [63-65]. Notably missing are discussions about translating general ethics and privacy principles into concrete guidelines in different national settings. Therefore, in accordance with guidance from our four ethics boards, we will advance the current state of the art in this domain by developing country-specific ethical guidelines for practice. There are some anticipated challenges and limitations of this research project. Although most of the data collection in each phase will take place in the language of each country, with Canada having two official languages, the overarching research process will be undertaken in English, and this may affect the capacity of team members, including older adults and caregivers, to fully participate. Communicating among different countries and time zones can be a challenge. Therefore, it is critical for this international team to have established solid lines of communication and formal institutional consortium agreements to ensure the success of the project. Second, despite the increasing computer literacy levels among older adults [66,67], some, especially those who are socioeconomically disadvantaged, do not have access to a computer with internet access and therefore will not be eligible to participate in Phase 2 of this project.

This protocol outlines an original approach to integrating mobility patterns and mobility experiences of older adults with memory problems into an e-DSI to improve their autonomy and ultimately inform SDM about housing options. The results will contribute to the development of technology that supports older adults’ autonomy and housing decisions in general. Furthermore, the international collaboration with end users can provide valuable insights into the intention to use technology for housing decisions and barriers to its use.

## Abbreviations

COORDINATEs: teChnology tO suppORt DecIsioN making about Aging aT homE
e-DSI: electronic decision support intervention
GRAMMS: Good Reporting of A Mixed Methods Study
NASSS: nonadoption, abandonment, scale-up, spread, and sustainability
SDM: shared decision making
VRW: virtual research workspace
